# Supplementary material for: DNA methylation as a mediator of HLA-DRB1*15:01 and a protective variant in multiple sclerosis
Source: Nat Commun. 2018 Jun 19;9:2397. doi: 10.1038/s41467-018-04732-5 (PMC6008330; doi:10.1038/s41467-018-04732-5)
Supplement: Supplementary file 1 — Supplementary Information [file 41467_2018_4732_MOESM1_ESM.pdf]

Supplementary Information

Kular L, Liu Y *et al*., DNA methylation as a mediator of HLA-DRB1*15:01 and a protective variant in Multiple Sclerosis

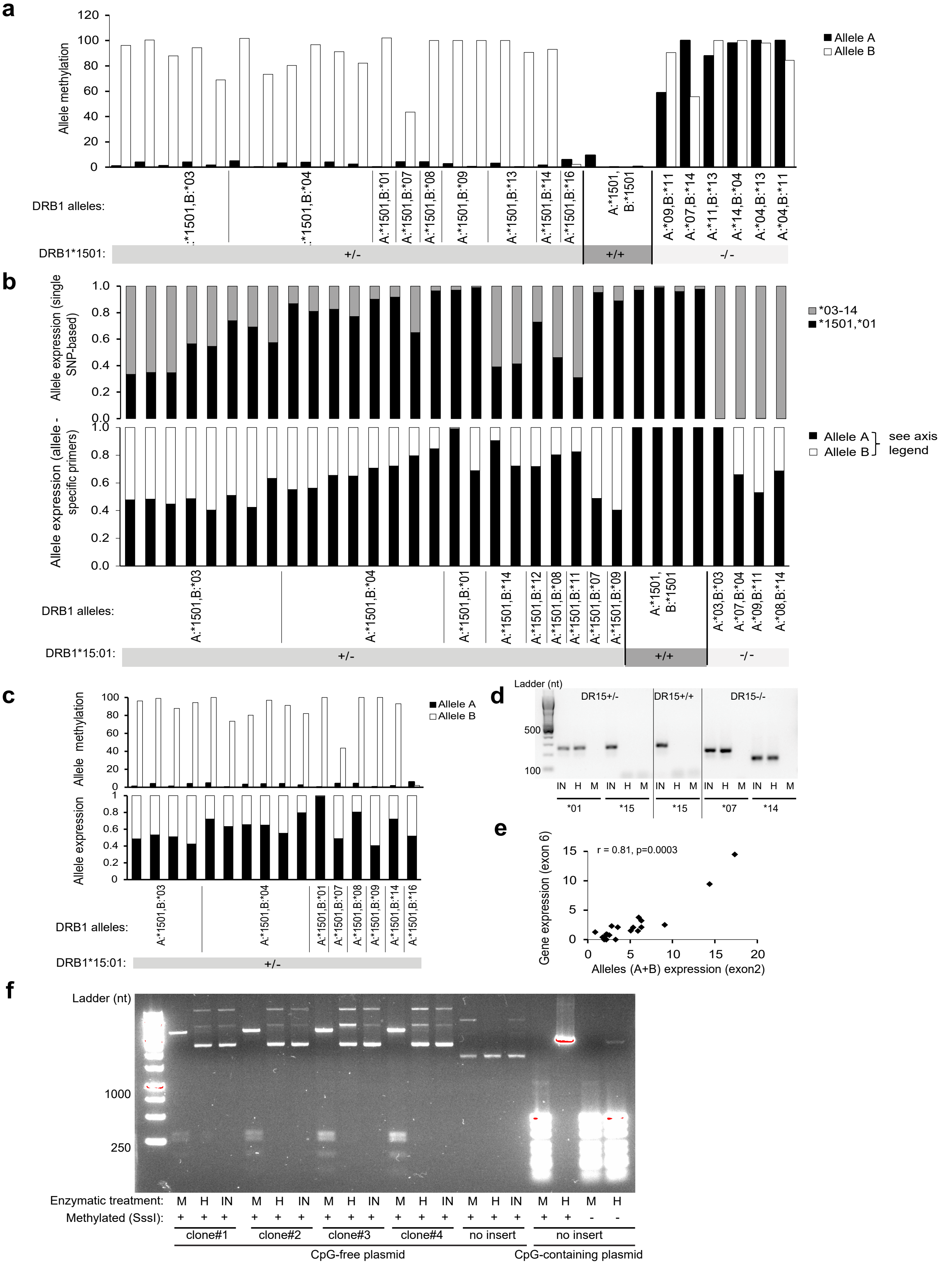

Supplementary Figure 1. HLA-DRB1 allele-specific methylation and expression in individuals heterozygous (+/-), homozygous (+/+) or non-carriers (-/-) of the DRB1*15:01 risk haplotype.

(**a**) Allele methylation of cg0603249 located in exon 2 using methyl-sensitive restriction enzyme (MSRE) followed by allele-specific qPCR (n=29). (**b**) Allele expression was assessed using two approaches in the same individuals: SNP-based qPCR distinguishing DRB1*15:01 or *01 from other alleles (*03-*14) and allele-specific primers (n=33). (**c**) Allele methylation and expression from the same individuals. (n=16) (**d**) Representative gel of allele methylation of cg0603249 using MSRE from three individuals carrying the following combination of HLA-DRB1 alleles *01/*15:01, *15:01/*15:01 and *07/*14. Products digested by the methyl-sensitive HpaII (H), the methyl-insensitive MspI (M) enzymes or undigested (input, IN) and amplified using allele-specific primers. (**e**) Correlation between expression of HLA-DRB1 alleles (allele A + allele B) using allele-specific primers located in exon 2 of the transcript and HLA-DRB1 expression quantified using other primers targeting exon 6 of the transcript, included is correlation coefficient r from Spearman test (n=21). (**a-c**): Allele A and B carried by each individual are shown below the X-axis. (**f**) Methylation of DRB1*15:01-vector used for in-vitro DNA methylation assay. Representative gel of methylated (by SssI enzyme) DRB1*15:01-containing (clone#1-4) and empty constructs treated with the methyl-insensitive MspI (M), the methyl-sensitive HpaII (H), enzymes or undigested (input, IN). An insert-free CpG-containing plasmid (pUC19) was used as control.

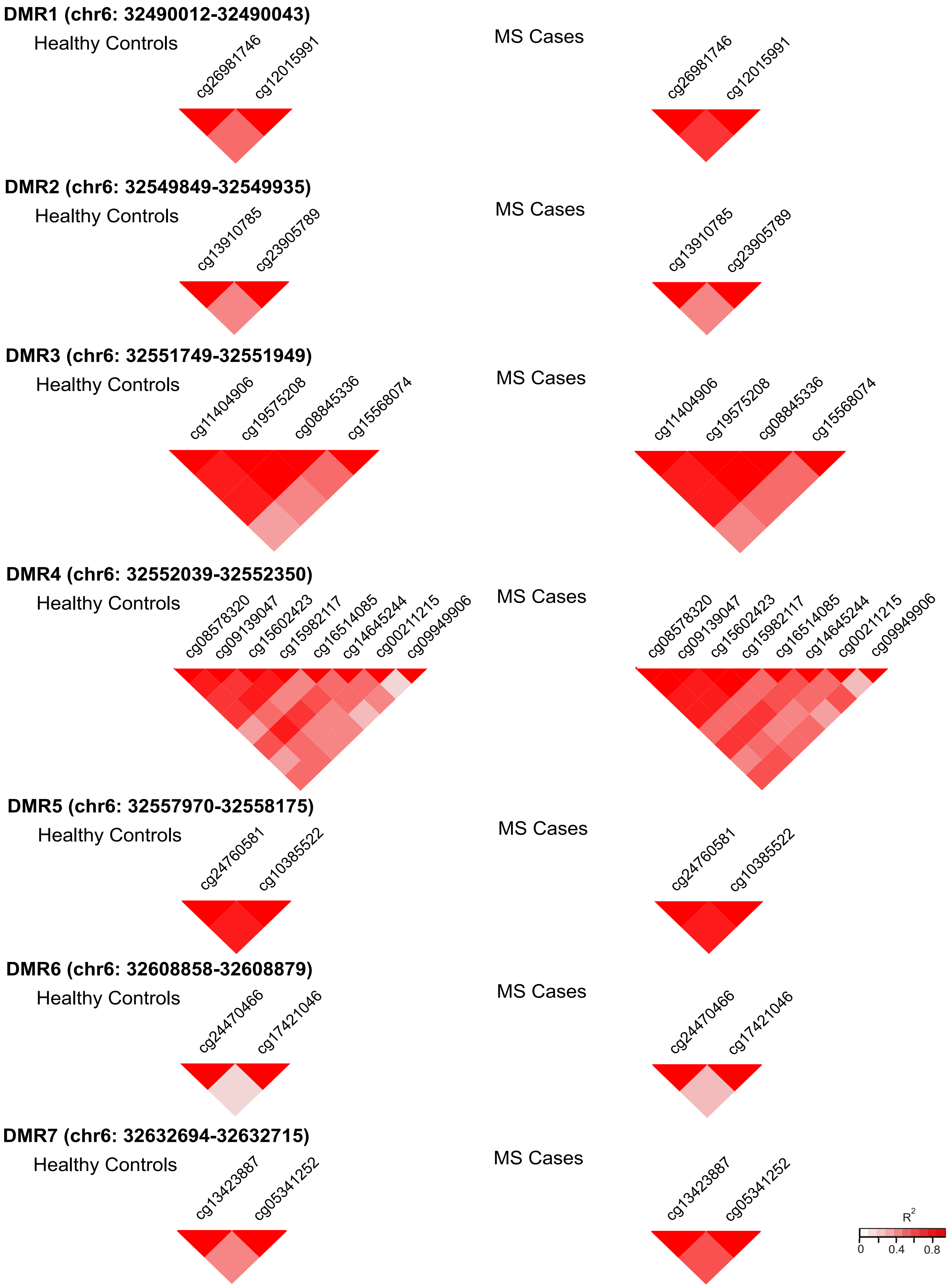

Supplementary Figure 2. Genetically controlled methylation clusters (GeMes).
The measure of the correlation coefficient of the methylation level, in 140 MS patients and 139 healthy controls, is shown graphically according to the shades of red; gray represents low r^2, and red represents high r^2.

a

DMR3			
ρ^2_{gx}	ρ^2_{gy}	$p_{Steiger}$	R
0.539	0.317	2.87E-03	6.533

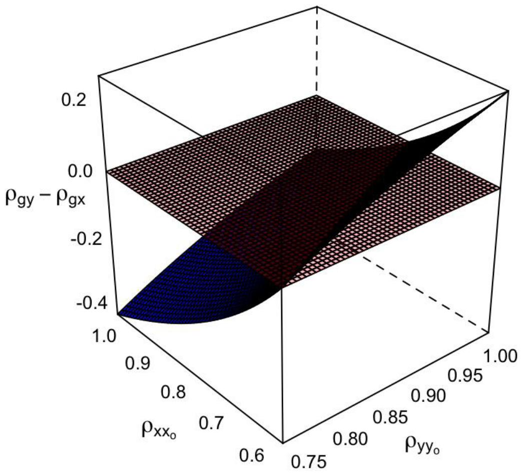

b

DMR4			
ρ^2_{gx}	ρ^2_{gy}	$p_{Steiger}$	R
0.563	0.317	8.65E-04	7.926

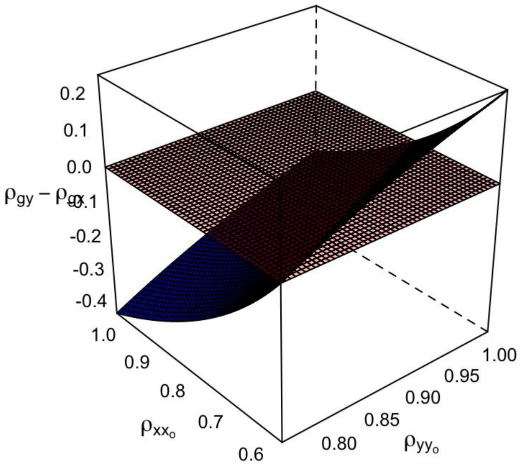

Supplementary Figure 3. MR Steiger and sensitivity analyses.

MR Steiger and sensitivity analyses for DMR3 (**a**) and DMR4 (**b**). The x and y axis represent different potential values of measurement error for the exposure (methylation) and the outcome (expression), respectively. The z axis shows the value of the MR Steiger test under measurement error (blue surface). When the correlation between the outcome and the variant (ρ_{gy}) is lower than the correlation between the exposure and the variant (ρ_{gx}), the values on the z axis are negative and the MR-Steiger test would infer the correct direction of the causality. Therefore, the MR-Steiger test supports the inferred direction of causality when the blue surface is below the black plane. Conversely, the test supports the wrong direction of causality when the blue surface is above the black plane. We can consider the positive and negative volumes bound by the blue and the black surface and calculate a reliability value (R) as the ratio between the negative and the positive volumes. The calculated R=6.5 and R=7.9 for DMR3 and DMR4, respectively, mean that it is R times more likely that the inferred direction of causality is correct (i.e., DNA methylation causes gene expression), compared to the opposite direction (gene expression causes methylation). Abbreviations: ρ^2_{gx}: the estimated variance explained in the exposure; ρ^2_{gy}: the estimated variance explained in the outcome; $p_{Steiger}$: the p value for inference of direction; R: the reliability or sensitivity ratio.

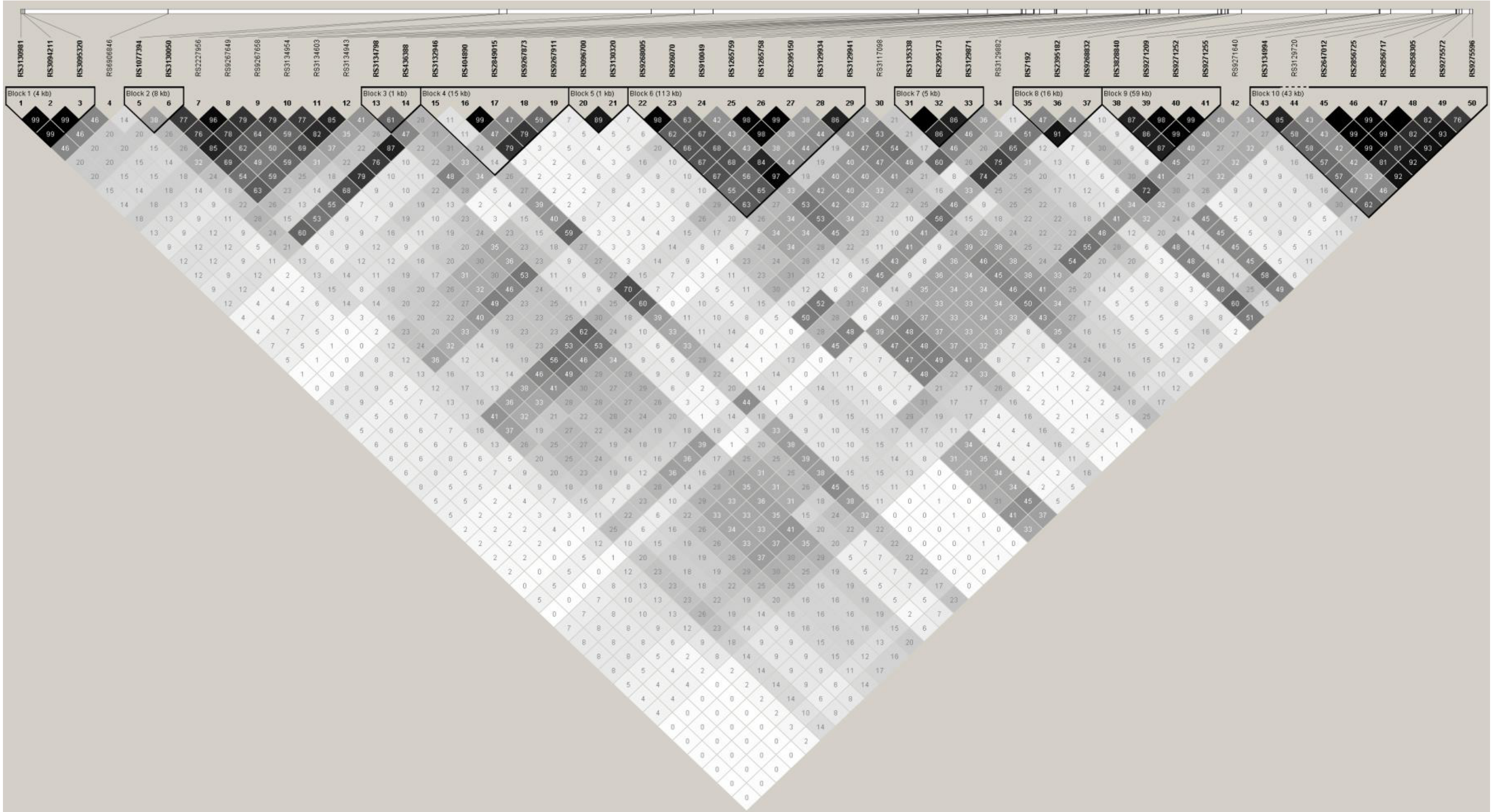

Supplementary Figure 4. R^2 for the 50 markers identified in the CIT analysis, block definition according to Garbriell et al.
Linkage disequilibrium analysis was performed in 728 MS cases and 625 controls from the Swedish population[5] using Haploview with standard settings.

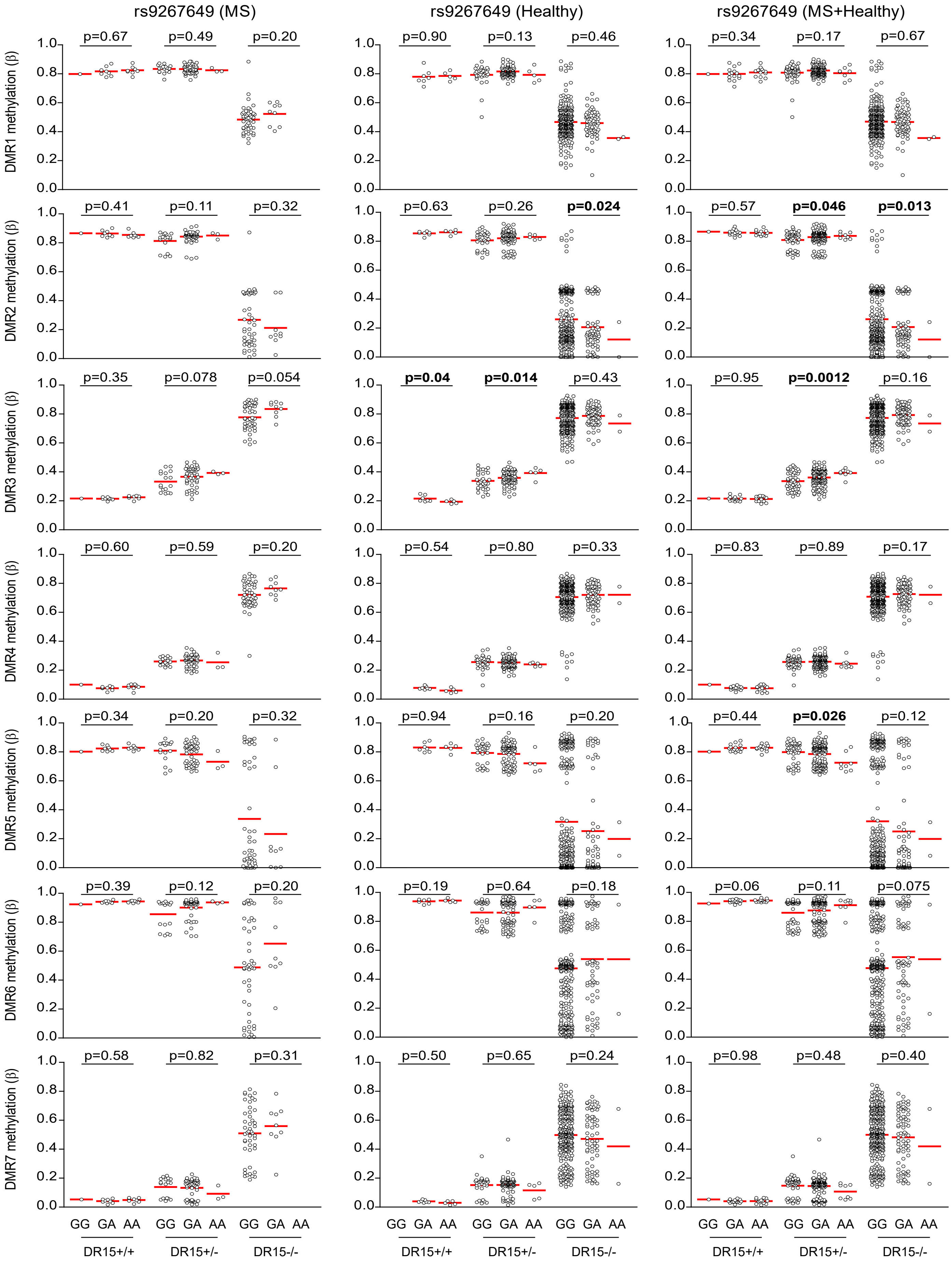

Supplementary Figure 5. Effect of rs9267649 and rs2227956 on the DNA methylation levels at in the HLA locus.
Association of rs9267649 and rs2227956 genotypes with methylation levels in the seven DMRs in the HLA locus was assessed using regression, after adjustment for confounders (age, sex, smoking status, hybridization batch and estimated differential cell counts, and disease status for analysis in cases and healthy controls together), in 140 MS patients and 472 healthy individuals stratified according to DRB1*15:01 risk variant, i.e. homozygous (DR15+/+), heterozygous (DR15+/-) and non-carrier (DR15-/-). P-value < 0.05 are highlighted in bold.
