# Supplementary material for: DNA methylation as a mediator of HLA-DRB1*15:01 and a protective variant in multiple sclerosis
Source: Nat Commun. 2018 Jun 19;9:2397. doi: 10.1038/s41467-018-04732-5 (PMC6008330; doi:10.1038/s41467-018-04732-5)
Supplement: Supplementary file 3 — Description of Additional Supplementary Files [file 41467_2018_4732_MOESM3_ESM.pdf]

Description of Additional Supplementary Files

File Name: Supplementary Data 1
Description: Description of cohorts.

File Name: Supplementary Data 2
Description: DNA methylation differences at HLA-DRB1 in monocytes.

File Name: Supplementary Data 3
Description: Primer sequences.

File Name: Supplementary Data 4
Description: SNPs that mediate risk for MS through changes in DNA methylation.

File Name: Supplementary Data 5
Description: DNA methylation differences at DMRs mediating genetic risk in sorted cells.

File Name: Supplementary Data 6
Description: Summary statistics for the meQTLs (exposure) and eQTLs (outcome) that were used in a two-sample Mendelian Randomization (MR).

File Name: Supplementary Data 7
Description: Association of CIT SNPs with MS after adjustment for the known MS risk variants in the HLA locus.

File Name: Supplementary Data 8
Description: RNA-seq data in PBMCs for HLA-DRB1 gene.
